# Long-Term Effects of Positive Psychotherapy Compared to Cognitive Behavior Therapy in Clinical Depression: An 18-Month Follow-Up Randomized Controlled Trial

**DOI:** 10.3390/healthcare14050692

**Published:** 2026-03-09

**Authors:** Elena Fischer, Linda Maria Furchtlehner, Raphael Schuster, Anton-Rupert Laireiter

**Affiliations:** 1 Department of Psychology, University of Salzburg, Hellbrunner Straße 34, 5020 Salzburg, Austria; anton.laireiter@plus.ac.at; 2 Neuromed Campus, Kepler University Hospital, Wagner-Jauregg-Weg 15, 4020 Linz, Austria; 3 Outpatient Center for Psychotherapy and Psychotherapy Research, Department of Psychology, University of Graz, 8010 Graz, Austria; raphael.schuster@uni-graz.at

**Keywords:** positive psychotherapy, long-term effects, group therapy, efficacy, positive psychology

## Abstract

**Highlights:**

**What are the main findings?**

- Positive Psychotherapy led to more sustained reductions in depressive symptoms than Cognitive Behavioral Therapy over an 18-month period.
- Long-term improvements in life satisfaction and positive psychological resources were greater in the PPT group.

**What are the implications of the main findings?**

- Strength-based interventions such as PPT may offer added long-term benefits beyond symptom-focused therapies in the treatment of depression.
- Focusing on well-being and positive resources can contribute to more durable treatment outcomes in depressive disorders.

**Abstract:**

**Background/Objectives**: Positive Psychotherapy (PPT) is an empirically supported treatment that directly targets positive resources and personal strengths as its primary logic. PPT is effective in amplifying happiness and well-being as an additional way to enhance positive mental health while also ameliorating symptoms of negative affect, especially in depression, anxiety disorders, and stress disorders. However, few studies have been conducted to investigate these effects in the long run. This study extends our previously published findings on the short-term efficacy of PPT by extending the follow-up period to 18 months and comparing its long-term effects with those of Cognitive Behavioral Therapy (CBT) within the same randomized controlled trial. **Methods**: Forty-nine out-patient participants with a DSM-IV diagnosis for depressive disorder (MDD, Dysthymia) were treated with 14 sessions of manualized PPT (*n* = 23) or CBT (*n* = 26) group therapy. In a randomized controlled two-center-study, questionnaires on depressive symptoms (BDI-II, MADRS, DHS), psychological distress (BSI), and well-being related outcomes (FS, PPTI, SWLS) were administered at baseline and 18-month follow-up. **Results**: Analyses using linear mixed models indicated significant differences in long-term treatment outcome for depressive symptoms (BDI-II, DHS, MADRS) and satisfaction with life (SWLS), depicting better outcomes for the PPT group. Between group effect sizes at 18-month follow-up were primarily in the middle range for all outcome measures, in favor of PPT. **Conclusions**: This study provides support for the long-term efficacy of PPT in the treatment of depression and improvement of positive resources.

## 1. Introduction

Depression is one of the most common and prevalent mental disorders, imposing high disease burden on affected individuals [1]. During the last decades, depression rates have been steadily increasing in adult and younger age groups [2,3] and there are many reasons to consider depression prevention and treatment as a primary mental health concern. Meanwhile, there are more than 200 studies examining the efficacy of various psychological treatments for depression. Cognitive Behavioral Therapy (CBT) is one of the most widely researched treatments for clinical depression and there is a great body of evidence for its efficacy and effectiveness [4]. Guided by empirical research, CBT focuses on teaching patients to modify their dysfunctional patterns in cognition (e.g., thoughts, beliefs, and attitudes), behavior, and emotional regulation [5], and then giving patients skills to reduce the risk of subsequent relapse. In accordance with the current state of traditional treatments for people with depression, CBT is based on a so-called deficit-oriented model of psychotherapy, where treatment is primarily concentrated on psychopathology, as well as deficits and dysfunctionality in thoughts, emotions, and behaviors. Establishing psychological resources has traditionally not been the focus of this kind of psychotherapy [6]. However, within Positive Psychology, mental health is conceptualized as not just the absence of symptoms but also the presence of positive feelings, including well-being. Over the past decade, the advent of Positive Psychology has yielded a lot of positive psychology interventions (PPIs), that is “[…] treatment methods or intentional activities that aim to cultivate positive feelings, behaviours or cognitions” [7] (p. 468), which are a promising approach to increasing well-being and satisfaction with life.

Positive Psychotherapy (PPT) is an alternative therapeutic approach based on the fundamental principles of positive psychology. Unlike standard interventions for depression, it assumes that effective treatment of the disorder does not require a focus on negative symptoms, but rather on directly building resources, strengths, and well-being [6]. The PPT protocol comprises 15 empirically validated practices organized into a cohesive treatment program primarily based on Seligman’s [8] PERMA model of well-being.

For PPT as a manualized treatment protocol [9], there are 20 studies demonstrating its effectiveness for enhancing well-being and reducing depressive symptoms compared to control or pre-treatment scores with medium to large effect sizes (for a summary see [9]). These studies have been conducted internationally and have addressed a variety of clinical populations, most of them in the group therapy format. Four of these studies have directly compared PPT with two well-established and well-researched treatments, Dialectical Behavior Therapy (DBT) and CBT. PPT was found to perform at least equally well or exceed the other treatments, particularly on well-being measures [10,11,12].

However, the research does not yet contain many studies that have directly examined follow-up outcome of any form of PPT [13]. Initially, group PPT was validated with 21 college students with mild to moderate depression, resulting in significantly fewer depressive symptoms compared to a control group that did not receive any intervention. This improvement remained stable for at least one year [6] (Study 1). Another study by Parks-Sheiner [14] compared 52 individuals who completed six PPT exercises online with a no-treatment control group of 69 individuals, resulting in lower levels of depression at the three- and six-month follow-ups. Similarly, Lü and Liu [15] compared a group PPT intervention (*n* = 16) with a no-treatment control group (*n* = 18) and explored the impact of positive affect on vagal tone when handling environmental challenges. PPT led to lower depression scores post-intervention that lasted for up to six months. Furthermore, Shoshani and Steinmetz [16] conducted a study in which they compared a school program involving PPT (*n* = 537) with a no-treatment control group (*n* = 501). After a two-year follow-up, students who participated in the PPT program showed significant reductions in general distress, anxiety, and depressive symptoms, as well as notable improvements in self-esteem, self-efficacy, and optimism. In another study, Ochoa et al. [12] assigned 126 consecutive female survivors of breast cancer with high levels of emotional distress to a group PPT program for cancer or to a waiting list control group. The PPT group obtained significantly better results after treatment than the control group, showing reduced distress, decreased post-traumatic symptoms, and increased post-traumatic growth. These gains were found to be sustainable for up to three and 12 months. We [17] conducted a randomized controlled trial comparing group PPT (*n* = 46) and CBT (*n* = 46) for individuals with depressive disorder, examining reductions in depressive symptoms and increases in happiness. PPT showed consistently moderate to high within- and between-group effect sizes compared to CBT, resulting in a higher remission rate of depression. Regarding feelings of happiness, PPT produced significantly higher scores on all outcome measures, with medium to large effect sizes, compared to CBT. These outcomes remained stable for up to six months for PPT, in contrast to CBT.

Investigating the long-term effects of positive psychotherapy (PPT), as reflected in an 18-month follow-up, is essential for understanding how psychological interventions contribute to lasting and sustainable improvements in well-being. Long-term outcomes also deepen our understanding of how therapeutic changes unfold over time, extending beyond the immediate relief of symptoms. Demonstrating long-term effectiveness would further support the allocation of time and resources to psychotherapy at a policy level. Therefore, examining the long-term effects of PPT is not only important from a methodological perspective, but also central to the field’s claims about the sustainable promotion of mental health.

### Aims and Research Questions

Studies on PPT outcome report decreases in negative affect and symptoms and increases in well-being that are sustainable for up to 12 months. However, research literature on the long-term outcomes of PPT is scarce, and further research is needed to expand on existing results to draw more profound conclusions. Moreover, the sample size of existing studies concerning long-term outcomes is rather small, so studies with larger sample sizes are required. As there are no prior studies on the long-term effects of PPT among individuals in Central Europe, this study focuses on a large sample of Austrian individuals with depressive disorders. Access to psychotherapeutic care in Central Europe is organized through public healthcare systems and is partly free. This is particularly relevant in this context, as the frequency and duration of access to therapy can influence its long-term effects.

The aim of the present study was to extend knowledge of the long-term clinical effectiveness of PPT versus CBT in reducing depressive as well as general psychological symptoms and in enhancing positive feelings of happiness in individuals with depressive disorders over an 18-month follow-up period. To this end, we conducted a randomized controlled trial in which we applied PPT in groups [6,9] and compared the results with those of the standard CBT intervention for treating depression [18], which was also applied in groups. The prevalence of mental health conditions often exceeds the available treatment resources, particularly in German-speaking countries, where group psychotherapy offers a viable alternative. There is also substantial evidence supporting the effectiveness of group therapy, showing outcomes comparable to those of individual psychotherapy [19]. Accordingly, we opted for a group setting, in line with previous studies on positive psychotherapy. We selected CBT as the control group because it currently represents the gold standard in the treatment of depression (e.g., [20]). The two main objectives of our study, both related to the 18-month follow-up period, are: Firstly, to investigate whether PPT is superior to CBT in enhancing positive resources, including well-being and happiness, and secondly, to explore whether PPT is superior to CBT in reducing depressive symptoms and psychological distress. Feelings of well-being and happiness were chosen as the primary outcomes because PPT focuses on enhancing psychological resources, including happiness and well-being. In line with this focus, self- and observer-rated depression and the individuals’ level of psychological distress were chosen as secondary outcomes.

This study is a long-term follow-up of two previously published pre–post and six-month follow-up studies comparing PPT and CBT [17,21]. Starting from identical levels of symptoms, PPT resulted in significantly lower levels of depressive and psychological distress symptoms at post-treatment and at the six-month follow-up, with consistently high effect sizes. In contrast, CBT resulted in smaller effects. Regarding well-being, including feelings of psychological well-being, life satisfaction, and flourishing, the PPT group resulted in significant improvements in all outcome measures, indicating medium-to-high effect sizes, and these improvements remained stable for up to six months. Furthermore, the findings revealed differences over time between the treatment conditions for the psychological well-being total score, two of its five subscales (positive emotions and engagement), the Flourishing Scale, and the Satisfaction with Life Scale, indicating superior outcomes for PPT following treatment.

Considering the results of our previous studies and the research into long-term outcomes in literature, we hypothesize that PPT will demonstrate superior long-term improvements in both outcome dimensions (well-being, depressive and psychological distress symptoms) compared to CBT, with these improvements remaining stable for up to 18 months.

## 2. Materials and Methods

### 2.1. Design

This two-center study is an 18-month follow-up of the previously reported randomized controlled trial, where participants (*n* = 92) were allocated either to PPT (*n* = 46) or CBT (*n* = 46) group therapies as two active conditions. Both treatments comprised 14 sessions of 2 h per week and were conducted according to the empirically supported PPT and CBT treatment manuals. The study’s interventions and data collection, containing baseline assessment (t1), post-intervention assessment (t2), a six-month follow-up, as well as an 18-month follow-up measurement, took place over a period of more than 2 years, beginning in May 2014 and finishing in October 2016. Findings from this large-scale study have been disseminated across multiple publications to allow for differentiated analyses of specific research questions and time points. While prior papers reported immediate post-treatment outcomes, the present paper reports exclusively on 18-month long-term follow-up data that has not been presented elsewhere.

### 2.2. Interventions

#### 2.2.1. Positive Psychotherapy (PPT)

The PPT group treatment followed the protocol developed by [9]. It is a manualized 14-session protocol, primarily for group therapy. Each session comprises a psychoeducational portion, in-session exercises, and group discussions. The most important parts, however, are the homework exercises, which are provided in a fixed sequence. Participants take at least one hour per week to complete these exercises. The goal is to promote clients’ understanding and practice of the basic principles of flourishing based on the PERMA-model. Some exercises run throughout the whole intervention (e.g., the “Three Good Things” application [22], where clients keep a daily journal about good things that happen during the day to counteract the negativity-bias [9]). Table 1 provides an overview of the session-by-session topics of the PPT condition.

#### 2.2.2. Cognitive Behavior Therapy (CBT)

In our study, CBT comprised the manualized 12-session-protocol for groups by Schaub et al. [18], which is a highly structured cognitive psycho-educative coping program for depression including interventions of psychoeducation and cognitive behavioral exercises based on a multidimensional functional concept of depression that comprises aspects of vulnerability, stressors, and coping strategies. Additionally, it deals with rewarding activities and includes information about treatment options, cognitive restructuring, and relapse prevention with weekly homework assignments. To make CBT conform to the 14-session-model of PPT, we added two extra sessions to the original manual: Based on the protocol’s stress-vulnerability model, one extra session deals with stress and stressors as possible causal agents of depression. Based on CBT principles, specific stress management strategies were imparted (cognitive, instrumental, and regenerative strategies, [23]) with a special focus on relapse prevention. “Savoring” is the topic of the second extra session, as in German-language countries, savoring is a very important part of CBT treatment of depression (e.g., [24]).

Thus, both therapies comprised 14 group sessions of 2 h per week (120 min) and each treatment was conducted according to its respective protocol. Table 2 provides an overview of the session-by-session topics of the cognitive behavioral group therapy and its extra sessions.

### 2.3. Participants and Procedure

**Figure 1:**
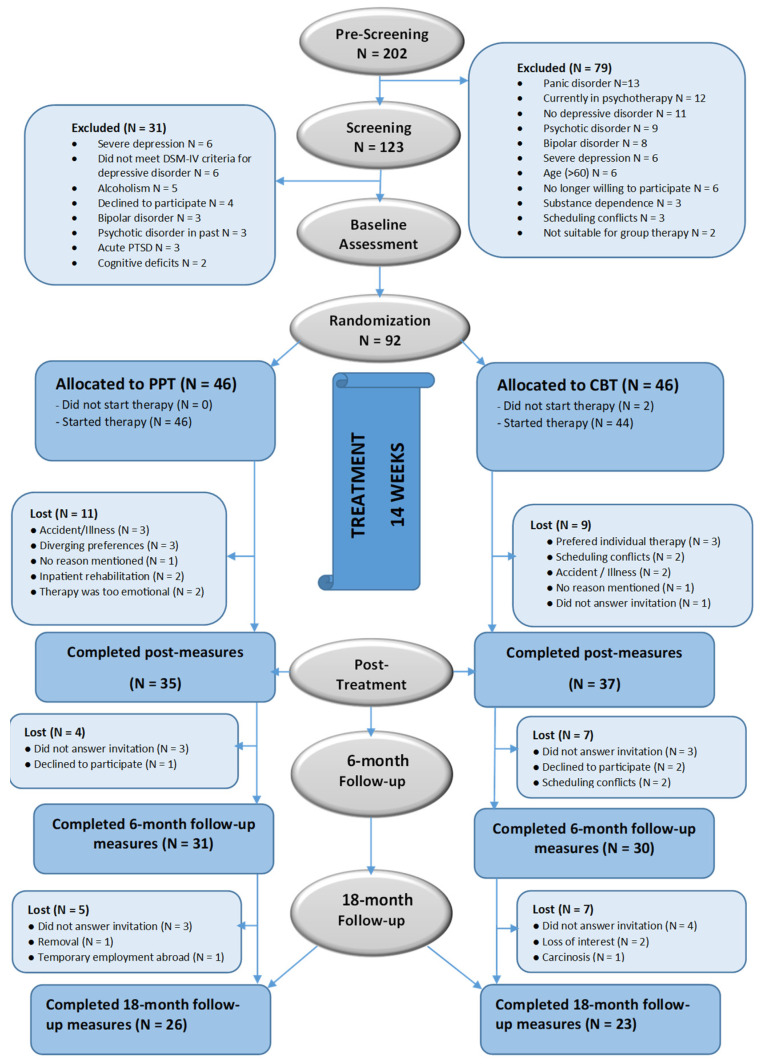
Flowchart of participants through each stage of the study. This figure has also appeared in our previous publications [17,21]. It is included here to provide context; however, the primary focus of this paper is the 18-month follow-up, which constitutes first-published results. Notes: PPT = Positive Psychotherapy; CBT = Cognitive Behavioral Therapy.

Sample size was calculated in advance using G*power analysis software 3.1 [25]. To detect small effects of Cohen’s d = 0.30 with a power of β = 0.80 and an α-level of 0.05, the estimated total sample size was *n* = 90. Before starting with the recruitment phase, approval for the present study was obtained by the federal ethics committee of Upper Austria, which was accepted by the research ethics board of the University of Salzburg. Participants were recruited nationally in two different treatment centers in Austria (two-center-study) with different kinds of acquisition. At the Outpatient Center of the Department of Psychology of the University of Salzburg, individuals were informed of the intended study through a university press release, a newspaper advertisement, and through an email to all students and employees registered or working at the university. At Linz, potential participants were recruited at the residential psychiatric hospital after their discharge. All subjects who applied for participation were selected using a three-step approach that is demonstrated in Figure 1. All potential participants were pre-screened first by phone or face-to-face in the hospital to check the basic criteria. Those who met basic criteria on diagnosis and demographic variables were then invited to an individual assessment session of approximately 3 h in length. During that time, detailed information about the ongoing study was provided and inclusion and exclusion criteria were checked using the German version of the Structured Clinical Interview for DSM-IV Axis I Disorders (SCID-I; [26]). Patients were eligible for the study if they (1) were between 18 and 60 years of age, (2) were having a mild to moderate major depressive disorder or dysthymia according to DSM-IV-TR [26], (3) spoke German sufficiently. Exclusion criteria were (1) currently undergoing psychotherapeutic or psychological treatment, (2) having a severe major depressive disorder, (3) current substance dependence, (4) current severe eating disorder, (5) current panic disorder, (6) hypomanic, manic, or bipolar disorder, or (7) psychotic disorders. In cases matching study criteria, signed informed consent was obtained from all participants; there was no compensation or payment for attendance. During participation, individuals were allowed to continue with their pharmacological treatments.

Of the 202 subjects registered for participation, a final 92 met the inclusion criteria. They completed baseline assessments and demographics through anonymous data collection. Subsequently, they were randomized to one of the treatment conditions (PPT or CBT), using a true random number service (www.random.org). Personnel who enrolled and those who assigned participants to the interventions had access to the random allocation sequence. Detailed information about participants’ flowcharts is shown in Figure 1.

Therapy was administered in small groups of six to seven people, and both treatments were applied in a single trainer setting. In total, seven different therapists were involved in this study. All of them were female clinical psychologists aged between 25 and 44 years. One in each condition (PPT, CBT) was a licensed clinical psychologist working in this field already for several years; the other five were in training for clinical psychology under supervision. Assignment of the group leaders was not random because of structural requirements of the respective institutions or group leaders’ individual preference for one or the other condition. Regardless of their current educational and experiential background, all trainers were intensively trained by two clinical psychologists who had sufficient clinical practice and experience in the respective treatments. Moreover, all group leaders were supervised weekly in the application of their respective protocols by their trainers. As additional control, every group leader had to protocol each session and the protocols were compared with the proposed manualized session process by independent raters. The therapy sessions were held in Salzburg at the Outpatient Center of the Department of Psychology and in Linz at the therapy facilities of Kepler Universitätsklinikum Linz. Both locations provided settings typical of outpatient therapy services.

### 2.4. Outcome Measures

The applied questionnaires comprised one observer-rated and six self-report measures. The assessments were carried out at the beginning and the end, and 6 months (follow-up I) and 18 months (follow-up II) after termination of treatment.

#### 2.4.1. Primary Outcomes

*Psychological well-being.* The Positive Psychotherapy Inventory (PPTI; [27]) is a PPT-related self-report questionnaire that was used to measure flourishing according to the PERMA or ‘flourishing’ model [8]. The German form was developed by translating the English items into German and retranslating into English by two independent, fully bilingual experts. One expert translated the scale from English into German, and the other conducted the back-translation. The final German version was reviewed and discussed within the research team. The PPTI consists of 25 items assessing Seligman’s five PERMA domains [8] with five items each (positive emotions, engagement, positive relationships, meaning, accomplishment) (for details see [9]). For each statement, the participant must choose the degree to which this item applies to them. Each answer is assigned a value from 1 (“not at all like me”) to 5 (“very much like me”), so that the total score ranges from 25 to 125, with higher scores indicating greater well-being. The internal consistency of the total scale is high, with α = 0.89. In the present study, the internal consistency of the total score was excellent, with α = 0.91 (baseline measurement). The internal consistency of the subscales ranged from acceptable to good (positive emotions: α = 0.78; engagement: α = 0.74; positive relationships: α = 0.86; meaning: α = 0.71; accomplishment: α = 0.77). At the 18-month follow-up, internal consistency was also excellent (total score: α = 0.93).

*Happiness/Flourishing*. The German form of the Flourishing Scale (FS-D; [28]) was used as a second measure to assess another facet of happiness and well-being. The scale consists of eight items, each being phrased in a positive direction using a seven-point Likert scale ranging from 1 (“strongly disagree”) to 7 (“strongly agree”), thus resulting in values between 8 and 56. Higher scores indicate a positive self-image and many psychological resources and strengths in important areas of functioning [29]. The internal consistency of the scale was high, with α = 0.87 (baseline measurement) and α = 0.92 (18-month follow-up).

*Satisfaction with Life* was measured with the Satisfaction with Life Scale (SWLS, [30,31]) consisting of five items using a seven-point Likert scale from 1 (“strongly disagree”) to 7 (“strongly agree”). In this study, the internal consistency was high, with α = 0.85 (baseline measurement) and α = 0.90 (18-month follow-up).

#### 2.4.2. Secondary Outcomes

*Depressive symptoms.* The revised 21-item Beck Depression Inventory-II (BDI-II, [32]; German adaptation by Hautzinger [33]) was used to assess depressive symptoms (item score 0–3; total score range: 0–63). According to the authors, the BDI-II has good clinical sensitivity and a high reliability ranging from α = 0.89 to α = 0.94. Internal consistency was high, with α = 0.89 (baseline measurement) and α = 0.93 (18-month follow-up).

*The Depression–Happiness Scale* (DHS, [34]) is a subjective measure for the continuum of depression to happiness, consisting of 25 items with an answer format from 0 (“never”) to 3 (“often”). The German form was developed by translating the English items into German and retranslating into English by two independent, fully bilingual experts. Internal consistency was excellent, with α = 0.92 (baseline measurement) and α = 0.93 (18-month follow-up).

*Severity of depressive episodes* was measured with the 10-item Montgomery–Asberg Depression Rating Scale (MADRS; [35,36]) as an observer-rated instrument. Item score ranges from 0 to 6, while total scores range from 0 to 60, higher scores indicating more severe depression. Internal consistency in this study was α = 0.84 (baseline measurement) and α = 0.87 (18-month follow-up).

*Psychological distress level and severity of symptoms* were assessed with the German form of the Brief Symptom Inventory (BSI) by Derogatis (German: [37]) comprising 53 symptoms rated on a five-point Likert scale. It contains nine primary symptom scales (somatization, depression, sensitivity, etc.) and three global indices of distress. In the present study, we used the most widely used Global Severity Index (GSI), which is designed to quantify a patient’s severity-of-illness as a single composite score. Internal consistency in the present study was excellent, with α = 0.93 (baseline measurement) and α = 0.92 (18-month follow-up).

### 2.5. Statistical Analysis

All statistical analyses were performed using IBM SPSS 25 (IBM Inc., Armonk, NY, USA) and R software version 2023.3.0.386 [38]. We included all participants in our analysis who completed the questionnaires, regardless of how many therapy sessions they attended or whether they completed the therapy. Potential differences in participants’ demographic and clinical characteristics at baseline were analyzed using Chi-square tests for categorical data and independent *t*-tests for continuous data. Cronbach’s Alpha was applied for reliability analyses at baseline. Estimated marginal means (*M*) and standard errors (*SE*) for each outcome measure were calculated at baseline as well as 18-month follow-up measurement.

Linear mixed models were performed to test long-term efficacy of treatment. Negative outcomes (depressive symptoms and psychological distress level) as well as positive outcomes (well-being and happiness) served as the dependent variables and their baseline levels as the covariates. Due to the study being carried out at two distinct locations, we incorporated study-site as a covariate in the models. Our analyses focused on a single dependent variable at a time, such as BDI-II, DHS, or SWLS. To address potential dependencies between participants in the repeated measures, random intercepts were incorporated in all models. To gain a more precise understanding of the changes in outcomes, we conducted pairwise comparisons with Tukey correction. This allowed us to examine differences within and between measurements and treatment groups.

We utilized the lmerTest package in RStudio Version 2024.12.1 [39] for conducting mixed-model analyses, while the Emmeans package was used for performing pairwise comparisons. To assess normality and homoscedasticity, the residual plots were visually examined, utilizing the sjPlot package in R [40]. The models were fitted using restricted maximum likelihood estimation (REML). Eta square was calculated for effect sizes (small effect η^2^ < 0.06; medium effect η^2^ = 0.06 to 0.14; large effect η^2^ ≥ 0.14; [41]).

## 3. Results

### 3.1. Sample Characteristics

No differences were found at baseline in demographic and clinical characteristics between both conditions (see Table 3). Similarly, no differences were found at baseline between both conditions in any of the outcome measures (see Table 4).

For the 92 participants at baseline, mean age was 40.66 years, most of them were female (*n* = 59; 64.1%), and educational level was rather high (see Table 3); 49 individuals (53%) completed assessments at 18-month follow-up. As demonstrated in Figure 1, 92 (100%) provided data at baseline, 77 (84%) at post-treatment, 61 (66%) at six-month follow-up, and 49 (53%) at 18-month follow-up. Attrition at 18-month follow-up was unrelated to treatment allocation (χ^2^ = 0.26, *df* = 1, *p* = 0.61). The average number of sessions attended for PPT was 10.70 ± 3.9 out of 14 and 10.70 ± 4.4 for CBT, indicating no significant differences in compliance. Equally, there was no differential dropout over time between both conditions (see Table 3).

### 3.2. Primary and Secondary Outcomes

Table 4 shows estimated marginal mean scores (and *SE*s) for baseline assessment and 18-month follow-up measurement for a first overview. In general, the mean levels of well-being related outcome measures increased over time in both conditions and the mean level of depressive and psychological distress symptoms decreased over time in both conditions.

The residual errors and random effects for all variables exhibit a normal distribution, indicating that the statistical assumptions for mixed-effects models are satisfied.

Significant Time × Group interactions emerged for the SWLS, DHS, BDI-II, and BSI (Table 5), showing that those in the PPT condition had lower levels of depressive symptoms (DHS, BDI-II, BSI) and more life satisfaction (SWLS) over time than those in the CBT condition. Superiority of PPT in these measures was associated with effect sizes of partial η^2^ > 0.02 at the 18-month follow-up, which can be categorized as small.

The interactions of Time × Group at 18-month follow-up were not significant for flourishing, PPTI total score and all subscales, and MADRS, indicating no significant differences in long-term treatment outcome between PPT and CBT (see Table 5).

We found significant effects of time for SWLS, flourishing, PPTI total score, positive emotions, engagement, DHS, MADS, BDI, and BSI, indicating significant improvements over time (see Table 5).

Pairwise comparisons revealed that all well-being related outcomes (satisfaction with life, flourishing, PPTI total score, the subscales positive emotions, engagement, relationship, meaning, and accomplishment) increased significantly and all depressive symptoms (DHS, MADRS, BDI-II) and psychological distress (BSI) decreased significantly in the PPT group from preintervention to 18-month follow-up (η^2^ = 0.19; *p* < 0.001; η^2^ = 0.14; *p* < 0.001; η^2^ = 0.16; *p* < 0.001; η^2^ = 0.17; *p* < 0.001; η^2^ = 0.14; *p* < 0.001; η^2^ = 0.04; *p* = 0.012; η^2^ = 0.07; *p* = 0.001; η^2^ = 0.07; *p* = 0.001; η^2^ = 0.16; *p* < 0.001; η^2^ = 0.28; *p* < 0.001; η^2^ = 0.29; *p* < 0.001; η^2^ = 0.19; *p* < 0.001, respectively; see also Table 4). In the CBT group, the subscale accomplishment increased significantly and the MADRS decreased significantly from preintervention to 18-month follow-up (η^2^ = 0.03; *p* = 0.05; η^2^ = 0.14; *p* < 0.001, respectively). We found between-group effects at the 18-month follow-up in well-being related outcomes (satisfaction with life, flourishing, PPTI total score, the subscales positive emotions, engagement, and meaning), depressive symptoms (DHS, MADRS, BDI-II), and psychological distress (BSI), indicating significant group differences at 18-month follow-up in favor of PPT (η^2^ = 0.08; *p* <.001; η^2^ = 0.04; *p* < 0.003; η^2^ = 0.04; *p* = 0.002; η^2^ = 0.06; *p* < 0.001; η^2^ = 0.04; *p* = 0.003; η^2^ = 0.02; *p* = 0.032; η^2^ = 0.07; *p* < 0.001; η^2^ = 0.03; *p* = 0.004; η^2^ = 0.12; *p* < 0.001; η^2^ = 0.11; *p* < 0.001, respectively).

There were no significant differences in any of the outcome variables when comparing the post-intervention measurement to the 18-month follow-up through pairwise comparisons. Thus, effects were stable over the 18-month follow-up period.

## 4. Discussion

This study contributes to the limited literature on the long-term superiority of PPT compared to CBT. It was designed as an RCT, targeting individuals with depressive disorders treated in two different areas in Austria. In the previous articles regarding the effectiveness of PPT compared to CBT, PPT was reported to be effective in treating depression with effects lasting for at least six months. Findings indicated significant superiority of PPT over time with consistently large within-effect sizes compared to the CBT condition, which showed minor effects. In terms of well-being related outcomes, PPT is effective in enhancing psychological well-being, happiness, and satisfaction with life for up to six months. In the present paper, long-term stability of these effects over a period of 18 months was addressed. Outcomes were available for 49 participants. The main results regarding this follow-up were that the mean levels of depressive and psychological distress symptoms decreased and the mean levels of well-being and happiness increased over time in both conditions. No significant differences were found between both conditions regarding the level of observer-rated depressive symptoms (MADRS), psychological well-being (PPTI), and flourishing (FS). However, importantly, PPT demonstrated superior long-term treatment outcomes regarding self-rated depressive (BDI-II, DHS) and psychological distress symptoms (BSI), and satisfaction with life (SWLS) compared to CBT. Between-group effect sizes favored PPT, ranging between η^2^ = 0.02 (PPTI) and η^2^ = 0.12 (BDI-II), which can be classified as small to medium.

Regarding the hypotheses established at the beginning of the study, it can be summarized that these were partially confirmed.

### 4.1. Results in Relation to the Literature

Within existing research, to the best of our knowledge, few studies to date have directly examined the long-term outcome of any form of PPT. Furthermore, most existing studies addressed only negative outcomes and did not include changes in positive resources [13]. Our findings not only confirm previous studies on the sustainability of PPT effects but extend existing evidence by demonstrating the superiority of PPT over CBT in long-term outcomes. Studies by Seligman et al. [6], Parks-Sheiner [14], Lü and Liu [15], and Ochoa et al. [12] show a reduction in depressive symptoms through PPT after 3 to 12 months. In contrast to these studies, other research has found no significant differences—only a trend—between CBT and PPT [13,42], which aligns with our findings regarding observer-rated depressiveness, psychological well-being, and happiness.

The present study is the first to evaluate the effectiveness of PPT over a long-term period of 18-month follow-up and compared with an active control-condition. Moreover, it is one of the few studies that have addressed long-term positive outcomes.

### 4.2. Limitations

However, there are some limitations in our study that restrict the generalizability of the results and may hinder final conclusions. First, although we started with a sufficiently large sample of 92 subjects, the 18-month sample must now be considered not particularly large. Despite a prolonged inclusion period and efforts to enhance the enrolment rate, we could not include more than 49 patients (53%) who completed assessments at 18-month follow-up. The most common reason for incomplete questionnaires was participants who stopped responding. To investigate possible biases due to missing data, we conducted independent *t*-tests. Across all outcome measures, demographics, and main diagnoses, we found no significant differences between the characteristics at baseline measurement of those who were lost to follow-up compared to those who completed the last questionnaires. Moreover, attrition was unrelated to treatment conditions. Therefore, it can be assumed that the absence or non-completion is random, and the principal study findings should hold true. However, the statistical power is reduced due to the missing data, which should be considered when interpreting the results.

Furthermore, given the number of outcomes analyzed, the issue of multiple testing must be acknowledged. This increases the risk of Type I errors, and findings should therefore be interpreted with caution. Another limitation is that, due to ethical reasons, we could not prevent participants from seeking any further psychotherapy during the 18-month follow-up period. As we did not pay heed to any additional treatment that participants made use of during the follow-up periods, our findings may be interpreted with caution. It could be that any further psychotherapy or other treatment alternative could have influenced the current findings in terms of overestimation of long-term treatment effects. Consequently, the extent to which differences in help-seeking behavior has an impact on follow-up outcome measures must be targeted in further studies. In this context, psychopharmacological treatment may also have influenced the results; however, when we included medication as a covariate, we did not observe any significant impact. Moreover, the results should be interpreted considering the non-random assignment of group leaders to the treatment conditions.

Another limitation is that we did not investigate variables specific to CBT, such as cognitive distortions (cognitive restructuring), behavioral patterns (behavioral activation, exposure to anxiety stimuli), or psychological flexibility. Future research should examine these outcomes in the context of PPT compared to CBT to better understand potential mechanisms of change and differential treatment effects. Also, the present paper relied primarily on self-report and (one) observer-rated measures. Future studies should incorporate additional assessment methods, such as behavioral tasks or physiological indicators (e.g., heart rate variability, cortisol) to obtain a more comprehensive and multi-method evaluation. Other limitations concerning the present study have been discussed in detail in previous papers ([17,21].

## 5. Conclusions

Our results demonstrate the superiority of PPT over CBT in reducing symptoms based on subjective evaluations 18 months after therapy. However, the present evidence regarding the enhancement of well-being is inconclusive. We successfully replicated the findings of the few existing studies on long-term outcome of PPT and extended the knowledge about long-term stability of PPT over a period of 18 months. However, research in this area remains at the very beginning stages. The current findings are promising and should encourage future work to further support the present results on the long-term stability of PPT. For instance, conducting a meta-analysis of existing follow-up studies could provide a more comprehensive and integrated understanding of PPT’s long-term effects. Also, future investigations should identify potential mediators and moderators that could shed light on the reasons behind these outcomes. For example, a likely reason that PPT outperforms CBT in the long term could be that PPT emphasizes the cultivation of positive emotions, strengths, and meaning, rather than solely focusing on symptom reduction.

From a practical perspective, our findings indicate that PPT may be a valuable alternative or complement to traditional CBT, particularly for patients who aim not only to alleviate symptoms but also to improve overall well-being and life satisfaction. Clinicians may benefit from moving beyond a model focused solely on symptom reduction and incorporate structured positive psychological interventions to foster long-term recovery, resilience, and life satisfaction in patients with depressive disorders. Greater emphasis could be placed on interventions that cultivate positive emotions, meaning, engagement, and interpersonal connectedness. Such approaches may help expand patients’ emotional and cognitive repertoire, especially in the later stages of treatment and in relapse prevention.

## Figures and Tables

**Table 1:** An Overview of Session-by-Session Description of Positive Psychotherapy *.

Session	Content
1. Orientation	Topic: The absence of positive resources in maintaining depression HW: Clients introduce themselves through a story in which they share a real-life event depicting them at their best
2. Character Strengths	Topic: Defining character strengths and discussion about their role in problem solving HW: Clients complete an online task (SSQ; Signature Strengths Questionnaire)
3. Signature Strengths	Topic: Computing Signature Strengths; discussion about goal setting to target specific problems or to cultivate more engagement HW: Clients frame specific goals into a concrete Signature Strengths Action Plan (SSAP)
4. Good & Bad Memories	Topic: Bad and bitter memories and how they perpetuate psychological distress; discussion about positive cognitive reappraisal strategies HW: Clients write about three bad memories and reflect on their impact in maintaining depression
5. Forgiveness	Topic: Forgiveness as a potential option to transform feelings of anger and bitterness HW: forgiveness letter
6. Gratitude	Topic: Gratitude as an enduring thankfulness; discussion about good and bad memories with an emphasis on gratitude. HW: gratitude letter
7. Mid-Therapy-Feedback Session	Topic: Signature Strengths Action Plan; follow-up of the forgiveness and gratitude assignments; necessary changes are made
8. Satisficing vs. Maximizing	Topic: Concepts of satisficing & maximizing and discussion about one’s own level HW: Clients identify and plan areas where they can benefit from satisficing
9. Hope, Optimism & Posttraumatic Growth	Topic: Optimism and hope in detail (clients think and write about times when important things were lost but other opportunities opened); potential growth from trauma is also explored and specific strategies are discussed to explore optimism in everyday life HW: Specific strategies to exercise optimism in everyday life
10. Positive Relationships	Topic: The role and importance of positive relationships in well-being Clients practise Active–Constructive Responding (ACR)—a strategy to foster positive relationship communication HW: Clients self-monitor for active–constructive opportunities
11. Signature Strengths of Others	Topic: Identification of character strengths of other family members HW: Clients ask family members to complete the SSQ online task and draw a family tree of strengths; discussion about family member’s signature strengths
12. Savoring	Topic: Savoring and its types and techniques with a savoring exercise; strategies to safeguard against adaption HW: Clients plan a savoring activity using specific techniques
13. Altruism	Topic: The therapeutic benefits of helping others HW: Clients plan to give a gift of time to someone using their signature strengths
14. The Full Life	Topic: Integration: The Full Life as the integration of positive emotions, engagement, positive relationship, meaning and accomplishment; discussion about therapeutic gains and experiences and ways to sustain positive changes are devised

Note: HW = Homework. * Based on [6]. Therapy was delivered as intended.

**Table 2:** Overview of session-by-session description of extended cognitive behavioral group therapy for depression *.

Topic	Session	Description
Psychoeducation	1	- Introduction - Organizational issues - Therapy concept - Dos and don’ts of how to behave in groups - Symptoms
	2	- Vulnerability stress model and vulnerability stress coping model
	3	- Medication and how antidepressants work - Other treatment strategies and psychotherapeutic approaches
Behavioral activation	4	- The vicious circle of depression - The depression spiral effect
	5	Positive activities and how to plan them
	6	- Importance of balance between positive activities and requirements/challenges (self-reinforcement)
Cognitive therapy	7	- Introduction of cognitive behavioral therapy - The cognitive triad of depression - The A-B-C theory
	8	- Cognitive distortions and how to change them - How to stop rumination
	9	- Typical depressive core beliefs and how they are influenced by automatically depressive thoughts
	10	- Identifying and changing depressive core beliefs
Relapse prevention	11	- Early warning signs of depression - Relapse prevention based on medication - How to deal with crises
	12	- How to manage depression - Follow-up care - Conclusion
Extra session 1	13	- Stress management: how depression is influenced by stress - Stressors and automatically running thoughts - Stress reaction (thoughts, emotions, physical reaction, behavior) - Stress management strategies (instrumental, cognitive, palliative–regenerative)
Extra session 2	14	- Savoring: how depression is influenced by a lack of savoring - Savoring strategies comprising all sensory perceptions (taste, olfaction, tactile sense, sense of sight, sense of hearing)

* Based on [18]. Therapy was delivered as intended.

**Table 3:** Demographic and clinical characteristics of the study samples at baseline.

	Treatment Group (PPT) (*n* = 46)	Control Group (CBT) (*n* = 46)	Total (*n* = 92)	Statistics
Demographic characteristics				
Gender: *n (%)*	Female: 31 (67.4)	Female: 28 (60.9)	Female: 59 (64.1)	χ^2^ (1, *n* = 92) = 0.43, *p* = 0.43, η^2^ = 0.005
Age: *Mean (SD)*	39.78 (11.53)	41.46 (13.35)	40.66 (12.4)	t (90) = −0.61, *p* = 0.54, η^2^ₚ = 0.004
Education: ≥ 9 years: *n (%)*	15 (32.6)	17 (36.9)	32 (34.8)	χ^2^ (3, *n* = 92) = 3.07, *p* = 0.038, η^2^ = 0.033
≥ 12 years: *n (%)*	21 (45.7)	16 (34.8)	37 (40.2)	
≥ 16 years *n (%)*	10 (21.7)	13 (28.3)	23 (25.0)	
Drop Out (Pre to post)	*n* = 11	*n* = 9	n = 20	χ^2^ (1, *n* = 92) = 0.26, *p* = 0.61, η^2^ = 0.003
Drop Out (Pre to 6-month follow-up)	*n* = 15	*n* = 16	n = 31	χ^2^ (1, *n* = 92) = 0.05, *p* = 0.83, η^2^ = 0.000
Drop Out (Pre to 18-month follow-up)	*n* = 20	*n* = 23	n = 43	χ^2^ (1, *n* = 92) = 0.39, *p* = 0.53, η^2^ = 0.004
Clinical characteristics of Diagnosis by SCID-I				χ^2^ (7, *n* = 92) = 5.57, *p* = 0.59, η^2^ = 0.057
Single Episode of Major depression				
mild: *n (%)*	1 (2.2)	2 (4.3)	3 (3.3)	
moderate: *n (%)*	10 (21.7)	6 (13.0)	16 (17.4)	
partial in remission: *n (%)*	1 (2.2)	3 (6.5)	4 (4.3)	
Recurrent Major depression				
currently mild: *n (%)*	1 (2.2)	4 (8.7)	5 (5.4)	
currently moderate: *n (%)*	24 (52.2)	23 (50.0)	47 (51.1)	
currently partial in remission: *n (%)*	1 (2.2)	0 (0.0)	1 (1.1)	
Dysthymia				
with double depression: *n (%)*	6 (13)	7 (15.2)	13 (14.1)	
without double depression: *n (%)*	2 (4.3)	1 (2.2)	3 (3.3)	
Severity of depression				
partial in remission: *n (%)*	2 (5.2)	3 (7.8)	5 (5.4)	
mild: *n (%)*	2 (5.2)	6 (15.8)	8 (8.7)	
moderate: *n (%)*	34 (89.5)	29 (76.3)	63 (68.5)	

Note. PPT: Positive Psychotherapy; CBT: Cognitive Psychotherapy; SCID-I: Structured Clinical Interview for DSM-IV Axis I Disorders.

**Table 4:** Descriptive statistics at baseline and 18-month follow-up among all study variables.

Applied Instruments	Preintervention		18-Month Follow-Up		Statistics at Baseline (Test of Between-Subjects Effects Between PPT and CBT)
	PPT	CBT	PPT	CBT	
	*M (SE)*	*M (SE)*	*M (SE)*	*M (SE)*	
SWLS	17.4 (0.67)	17.3 (0.67)	23.0 (0.72) _a,b_	18.3 (0.72)	*F* (1, 90) = 0.00, *p* = 0.95, η^2^_p_ = 0.000
FS	34.6 (0.93)	34.3 (0.93)	41.3 (1.00) _a,b_	37.1 (1.01)	*F* (1, 90) = 0.89, *p* = 0.35, η^2^_p_ = 0.010
PPTI_total	77.9 (1.48)	77.4 (1.49)	89.2 (1.60) _a,b_	82.1 (1.60)	*F* (1, 90) = 0.60, *p* = 0.44, η^2^_p_ = 0.007
PPTI_P	13.9 (0.42)	13.6 (0.42)	17.5 (0.45) _a,b_	14.9 (0.45)	*F* (1, 90) = 3.38, *p* = 0.07, η^2^_p_ = 0.036
PPTI_E	16.6 (0.40)	16.6 (0.40)	19.3 (0.43) _a,b_	17.5 (0.43)	*F* (1, 90) = 0.00, *p* = 1.00, η^2^_p_ = 0.000
PPTI_R	17.5 (0.40)	17.5 (0.40)	19.1 (0.44) _b_	18.1 (0.44)	*F* (1, 90) = 0.08, *p* = 0.78, η^2^_p_ =0.001
PPTI_M	13.9 (0.36)	13.7 (0.36)	15.6 (0.38) _a,b_	14.5 (0.39)	*F* (1, 90) = 1.39, *p* = 0.24, η^2^_p_ = 0.015
PPTI_A	15.9 (0.38)	15.9 (0.38)	17.6 (0.41) _b_	17.1 (0.41) _b_	*F* (1, 90) = 0.13, *p* = 0.72, η^2^_p_ = 0.001
DHS	32.9 (1.90)	32.7 (1.91)	47.8 (2.05) _a,b_	35.0 (2.06)	*F* (1, 90) = 0.05, *p* = 0.83, η^2^_p_ = 0.001
MADRS	21.3 (1.07)	22.01 (1.10)	9.10 (1.15) _a,b_	13.95 (1.19) _b_	*F* (1, 90) = 0.85, *p* = 0.36, η^2^_p_ = 0.010
BDI-II	24.85 (1.33)	25.18 (1.33)	9.99 (1.43) _a,b_	21.48 (1.43)	*F* (1, 90) = 0.24, *p* = 0.62, η^2^_p_ = 0.003
BSI	1.14 (0.06)	1.16 (0.06)	0.60 (0.07) _a,b_	1.11 (0.07)	*F* (1, 90) = 0.58, *p* = 0.45, η^2^_p_ = 0.006

Note. ^a^: indicates significant difference between Positive Psychotherapy (PPT) and Cognitive Psychotherapy (CBT) at the same time point (*p* < 0.05); ^b^: indicates significant difference estimated marginal mean at 18-month follow-up is significantly higher than at preintervention (*p* < 0.05). SWLS: Satisfaction with Life Scale; FS: Flourishing Scale; PPTI_total: Positive Psychotherapy Inventory_total score; PPTI_P: Positive Psychotherapy Inventory_Positive emotions; PPTI_E: Positive Psychotherapy Inventory_Engagement; PPTI_R: Positive Psychotherapy Inventory_Positive relationships; PPTI_M: Positive Psychotherapy Inventory_Meaning; PPTI_A: Positive Psychotherapy Inventory_Accomplishment; DHS: Depression–Happiness Scale; MADRS: Montgomery–Asberg Depression Rating Scale; BDI-II: Beck Depression Inventory-II; BSI: Brief Symptom Inventory. *M* = estimated marginal means; *SE* = standard error.

**Table 5:** Parameter estimates for linear mixed models of all study variables including interaction terms.

Fixed Effects (Intercepts, Slopes)	Est	*SE*	*t*	*p*	95% CI LL	95% CI UL	Partial *η^2^*
SWLS							
Intercept	2.55	2.31	1.10	0.271	−1.98	7.09	0.00
Treatment	0.12	1.20	0.10	0.923	−2.23	2.46	0.00
Time	**2.91**	0.70	4.15	<0.001	1.54	4.28	0.07
Site	−0.42	0.75	−0.56	0.575	−1.88	1.04	0.00
SWLS_T1	**0.86**	0.06	15.16	<0.001	0.75	0.97	0.72
Treatment × Time	**−1.19**	0.45	−2.66	0.008	−2.07	−0.31	0.03
FS							
Intercept	**8.77**	3.51	2.50	0.013	1.90	15.64	0.03
Treatment	−1.04	1.66	−0.63	0.530	−4.30	2.21	0.00
Time	**2.35**	0.97	2.44	0.016	0.46	4.25	0.03
Site	−0.78	1.03	−0.76	0.451	−2.80	1.24	0.00
FS_T1	**0.80**	0.06	14.06	<0.001	0.69	0.91	0.69
Treatment × Time	−0.34	0.62	−0.56	0.579	−1.56	0.87	0.00
PPTI_total							
Intercept	**14.82**	6.24	2.37	0.019	2.58	27.06	0.03
Treatment	−1.54	2.65	−0.58	0.563	−6.74	3.66	0.00
Time	**4.03**	1.54	2.62	0.009	1.01	7.05	0.03
Site	0.06	1.64	0.04	0.967	−3.15	3.29	0.00
PPTI_total_T1	**0.82**	0.05	15.28	<0.001	0.72	0.93	0.72
Treatment × Time	−0.80	0.99	−0.81	0.416	−2.73	1.13	0.00
PPTI_P							
Intercept	**4.21**	1.56	2.69	0.008	1.14	7.27	0.03
Treatment	−0.88	0.77	−1.14	0.253	−2.38	0.62	0.00
Time	**1.13**	0.46	2.44	0.015	0.22	2.03	0.03
Site	−0.41	0.42	−0.98	0.330	−1.24	0.41	0.01
PPTI_P_T1	**0.81**	0.06	14.43	<0.001	0.70	0.92	0.69
Treatment × Time	−0.12	0.30	−0.40	0.693	−0.70	0.46	0.00
PPTI_E							
Intercept	**4.08**	1.55	2.64	0.009	1.05	7.12	0.04
Treatment	−0.18	0.70	−0.26	0.792	−1.55	1.19	0.00
Time	**1.26**	0.40	3.14	0.002	0.47	2.04	0.04
Site	0.59	0.47	1.26	0.210	−0.32	1.50	0.02
PPTI_E_T1	**0.69**	0.06	10.76	<0.001	0.57	0.82	0.55
Treatment × Time	−0.35	0.26	−1.36	0.174	−0.85	0.15	0.00
PPTI_R							
Intercept	**3.61**	1.53	2.37	0.019	0.62	6.60	0.03
Treatment	0.14	0.71	0.19	0.846	−1.26	1.53	0.00
Time	0.77	0.42	1.85	0.065	−0.04	1.59	0.01
Site	0.21	0.42	0.50	0.616	−0.61	1.03	0.00
PPTI_R_T1	**0.75**	0.04	17.09	<0.001	0.67	0.84	0.77
Treatment × Time	−0.26	0.27	−0.96	0.338	−0.78	0.27	0.00
PPTI_M							
Intercept	**3.67**	1.33	2.75	0.007	1.05	6.28	0.04
Treatment	−0.51	0.63	−0.80	0.424	−1.74	0.73	0.00
Time	0.39	0.37	1.06	0.292	−0.34	1.12	0.00
Site	−0.17	0.37	−0.47	0.637	−0.89	0.54	0.00
PPTI_M_T1	**0.80**	0.05	16.98	<0.001	0.70	0.89	0.77
Treatment × Time	0.00	0.24	0.02	0.988	−0.46	0.47	0.00
PPTI_A							
Intercept	**3.88**	1.48	2.63	0.009	0.99	6.77	0.04
Treatment	−0.11	0.67	−0.17	0.869	−1.41	1.19	0.00
Time	0.52	0.38	1.37	0.172	−0.22	1.26	0.00
Site	0.32	0.43	0.74	0.460	−0.52	1.16	0.00
PPTI_A_T1	**0.74**	0.06	13.20	<0.001	0.63	0.84	0.64
Treatment × Time	−0.12	0.24	−0.49	0.623	−0.59	0.35	0.00
DHS							
Intercept	5.52	6.42	0.86	0.391	−7.07	18.10	0.00
Treatment	−0.49	3.41	−0.14	0.886	−7.16	6.19	0.00
Time	**8.40**	2.00	4.19	<0.001	4.47	12.33	0.07
Site	2.11	2.05	1.03	0.305	−1.90	6.12	0.01
DHS_T1	**0.69**	0.06	10.96	<0.001	0.57	0.81	0.56
Treatment × Time	**−3.06**	1.28	−2.39	0.018	−5.58	−0.55	0.02
MADRS							
Intercept	5.68	4.46	1.27	0.200	−3.05	14.42	0.00
Treatment	0.54	2.08	0.26	0.790	−3.54	4.62	0.00
Time	**−5.07**	1.25	−4.04	<0.001	−7.52	−2.61	0.07
Site	1.90	1.28	1.49	0.140	−0.60	4.40	0.02
MADS_T1	**0.60**	0.07	8.52	<0.001	0.46	0.74	0.45
Treatment × Time	1.26	0.80	1.56	0.120	−0.32	2.84	0.01
BDI							
Intercept	7.62	5.31	1.43	0.153	−2.80	18.03	0.01
Treatment	1.41	2.47	0.57	0.568	−3.43	6.26	0.00
Time	**−6.55**	1.46	−4.48	<0.001	−9.41	−3.69	0.08
Site	−0.53	1.58	−0.33	0.739	−3.63	2.57	0.00
BDI_T1	**0.70**	0.07	10.00	<0.001	0.56	0.83	0.53
Treatment × Time	**1.97**	0.94	2.11	0.036	0.14	3.80	0.02
BSI							
Intercept	0.15	0.24	0.63	0.530	−0.32	0.62	0.00
Treatment	0.09	0.11	0.75	0.452	−0.14	0.31	0.00
Time	**−0.25**	0.07	−3.73	<0.001	−0.39	−0.12	0.06
Site	0.04	0.07	0.59	0.556	−0.10	0.19	0.00
BSI_T1	**0.75**	0.06	13.01	<.001	0.64	0.86	0.65
Treatment × Time	**0.09**	0.04	2.06	0.041	0.00	0.17	0.02

Note: Est = unstandardized estimates; *SE* = standard error; CI = confidence interval; LL = lower limit; UL = upper limit; SD = standard deviation; significant coefficients are in bold (*p* = 0.05); T1 = baseline. SWLS: Satisfaction with Life Scale; FS: Flourishing Scale; PPTI_total: Positive Psychotherapy Inventory_total score; PPTI_P: Positive Psychotherapy Inventory_Positive emotions; PPTI_E: Positive Psychotherapy Inventory_Engagement; PPTI_R: Positive Psychotherapy Inventory_Positive relationships; PPTI_M: Positive Psychotherapy Inventory_Meaning; PPTI_A: Positive Psychotherapy Inventory_Accomplishment; DHS: Depression–Happiness Scale; MADRS: Montgomery–Asberg Depression Rating Scale; BDI-II: Beck Depression Inventory-II; BSI: Brief Symptom Inventory.

## Data Availability

The data and corresponding analyses are available from the Open Science Framework (OSF): https://osf.io/etgbh/overview?view_only=9d406b4f793c46fd870f728c1aed664f (accessed on 24 December 2025).
